# Supplementary material for: Transcriptomic Insights into Mechanisms of Early Seed Maturation in the Garden Pea (Pisum sativum L.)
Source: Cells. 2020 Mar 23;9(3):779. doi: 10.3390/cells9030779 (PMC7140803; doi:10.3390/cells9030779)

Sprint−2(10) vs Zhongw

Global Summary

%DE = 0.93

genes with fdr < 0.2 = 578 (286 + / 292 −)

genes with fdr < 0.1 = 535 (263 + / 272 −)

genes with fdr < 0.05 = 509 (250 + / 259 −)

genes with fdr < 0.01 = 389 (185 + / 204 −)

genes in genesets = 0

<FC> = 0

<t−score> = −9.59

<p−value> = 0

<fdr> = 0.08

Portrait

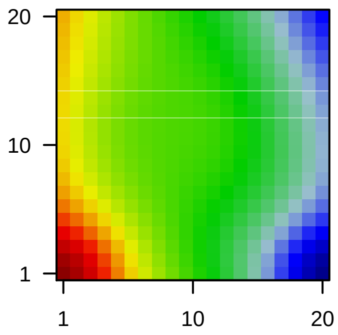

Top 100 DE genes

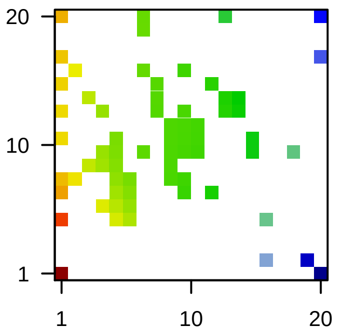

Global Genelist

Rank	ID	log(FC)	p−value	fdr	Metagene	Description
1	TRINITY_DN1	−19.01	1e−11	0.00000120	x 1	
2	TRINITY_DN1	0	3e−08	0.00000111	x 10	
3	TRINITY_DN3	−1.05	6e−08	0.00000220	x 17	
4	TRINITY_DN4	5.72	9e−08	0.0000021	x 1	
5	comp4201_c0	−0.03	1e−07	0.0000029	x 8	
6	TRINITY_DN6	0.48	2e−07	0.0000031	x 13	
7	TRINITY_DN1	0	3e−07	0.0000038	x 14	
8	TRINITY_DN7	0.33	4e−07	0.0000031	x 8	
9	TRINITY_DN6	0.25	5e−07	0.0000034	x 6	
10	TRINITY_DN1	−0.01	5e−07	0.0000039	x 11	
11	TRINITY_DN4	0.09	5e−07	0.0000065	x 7	
12	TRINITY_DN1	−0.02	8e−07	0.0000069	x 9	
13	TRINITY_DN2	0.11	1e−06	0.0000063	x 14	
14	TRINITY_DN6	0.07	1e−06	0.0000065	x 11	
15	TRINITY_DN1	−0.2	1e−06	0.00000618	x 10	
16	TRINITY_DN3	0	1e−06	0.0000068	x 14	
17	TRINITY_DN4	0	1e−06	0.0000069	x 11	
18	comp40971_c	−1.2	2e−06	0.00000616	x 2	
19	TRINITY_DN3	0	2e−06	0.0000079	x 11	
20	TRINITY_DN7	0.07	2e−06	0.0000075	x 8	

p−values

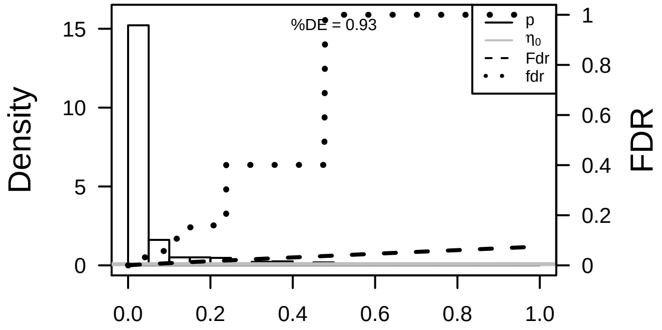

Supplement: Supplementary file 1 [file cells-09-00779-s001.zip › Supplementary materials/FileS3.zip!/FileS2/Hormones - Results/Summary Sheets - Differences/Reports/Sprint.2.10..vs.Zhongwan.6.10..pdf]
